# Mini-Tn*7* vectors for stable expression of diguanylate cyclase PleD* in Gram-negative bacteria

**DOI:** 10.1186/s12866-015-0521-6

**Published:** 2015-09-29

**Authors:** Lorena Romero-Jiménez, David Rodríguez-Carbonell, María Trinidad Gallegos, Juan Sanjuán, Daniel Pérez-Mendoza

**Affiliations:** Departamento Microbiología del Suelo y Sistemas Simbióticos, Estación Experimental del Zaidín, Consejo Superior de Investigaciones Científicas (CSIC), Granada, Spain

**Keywords:** c-di-GMP, mini-Tn*7*, Signal transduction, Biofilms, Exopolysaccharide production, Bacterial motility, Plasmid stability

## Abstract

**Background:**

The cyclic diguanylate (c-di-GMP) is currently considered an ubiquitous second messenger in bacteria that influences a wide range of cellular processes. One of the methodological approaches to unravel c-di-GMP regulatory networks involves raising the c-di-GMP intracellular levels, e.g. by expressing a diguanylate cyclase (DGC), to provoke phenotypic changes.

**Results:**

We have constructed mini-Tn*7* delivery vectors for the integration and stable expression of the pleD* gene encoding a highly active DGC, which can be used to artificially increase the intracellular levels of c-di-GMP in Gram negative bacteria. The functionality of these new vectors has been validated in several plant-interacting α- and γ-proteobacteria. Similarly to vector plasmid-borne pleD*, the genome-borne mini-Tn*7*pleD* constructs provide significant increases in intracellular c-di-GMP, provoking expected phenotypic changes such as enhanced polysaccharide production, biofilm formation and reduced motility. However, the mini-Tn*7*pleD* constructs resulted far more stable in the absence of antibiotics than the plasmid-based pleD* constructs. Furthermore, we have also implemented an inducible system to modulate pleD* expression and intracellular c-di-GMP rises “on demand”.

**Conclusions:**

mini-Tn*7*pleD* constructs are very stable and are maintained during bacterial free-living growth as well as during interaction with eukaryotic hosts, in the absence of selective pressure. This high stability ensures experimental homogeneity in time and space with regard to enhancing c-di-GMP intracellular levels in bacteria of interest.

**Electronic supplementary material:**

The online version of this article (doi:10.1186/s12866-015-0521-6) contains supplementary material, which is available to authorized users.

## Background

The cyclic diguanylate (c-di-GMP) was discovered only 27 years ago as an allosteric activator of bacterial cellulose synthase, but is currently considered an ubiquitous second messenger in bacteria that influences a wide range of cellular processes, including flagellum-mediated motility, cell cycle and exopolysaccharide (EPS) biosynthesis, as well as bacterial virulence [1]. c-di-GMP signalling systems are generally composed of three major constituents: diguanylate cyclases (DGCs, synthesize c-di-GMP from two GTP molecules), phosphodiesterases (PDEs, degrade c-di-GMP) and c-di-GMP-binding effectors [2, 3]. In general, the GGDEF domain of DGCs and the EAL or HD-GYP domains of PDEs are responsible for DGC and PDE activities, respectively, and balanced control of these opposite activities determines c-di-GMP homeostasis within the cell [3]. Genome analyses have revealed that the number of proteins with DGC and PDE domains is variable, highlighting the ability of bacteria to adapt to different habitats, the range of environmental stimuli perceived and/or the cellular functions affected by them. For instance, free-living bacteria with complex environmental lifestyles and co-evolutionary relationships with eukaryotes possess far more c-di-GMP-metabolizing enzymes than obligate parasites [1, 4, 5]. In that sense, cellular levels of c-di-GMP can be viewed as integral outputs of bacterial sensory systems that perceive various biotic and abiotic conditions. c-di-GMP translates input signals into the modulation of cellular behaviours by binding to diverse effector molecules, which so far include specific c-di-GMP receptor proteins (with PilZ, GIL or degenerate GGDEF/EAL domains), c-di-GMP-binding transcription factors, and RNA motifs (riboswitches) [2, 3, 6–9]. The large diversity of effector elements is indicative of the c-di-GMP regulation at multiple levels: transcriptional, posttranscriptional and posttranslational [1, 2, 10–12]. However, the multiplicity of DGCs and PDEs contrast with the comparatively few albeit functionally diverse c-di-GMP receptors/effectors identified so far, suggesting the existence of yet many unknown effectors. Thus, additional approaches besides genomics and bioinformatics need to be implemented to uncover novel c-di-GMP regulation pathways and targets, particularly in bacteria with complex lifestyles.

One such approach involves artificial modification of the c-di-GMP economy, by either overexpressing a DGC or a PDE, to identify associated phenotypic changes. In a recent work, the c-di-GMP levels of several plant-interacting bacteria were increased by expressing the DGC PleD* [13]. The pleD* gene expressed from a plasmid vector (pJBpleD*) altered a number of free-living phenotypes, as well as the interaction with their plant hosts. pleD* overexpression has also proven to be useful for uncovering novel and otherwise cryptic EPSs in different bacteria [14, 15]. Although the pleD* plasmid was certainly a powerful tool, its use was limited by its low stability under non selective conditions (i.e., absence of antibiotics), which often led to rapid loss, particularly in association with plants [13]. To overcome this limitation, we have constructed new vehicles based on the Tn*7* transposon [16, 17] for genome integration of the pleD* gene. Tn*7* inserts into a specific site called *att*Tn*7* and with a determined orientation [18, 19]. Most bacteria possess a single *att*Tn*7* site [20–22], which is frequently localized downstream of the *glmS* gene (encoding glucosamine-fructose-6-phosphate aminotransferase), and where transposon insertions do not usually affect bacterial fitness. Indeed, Tn*7* transposon derivatives have been widely used to introduce genes into bacterial chromosomes and insertions of Tn*7* transposon have been successfully obtained in a plethora of different bacteria [16]. Stability and efficacy of the pleD* mini-transposons have been tested in plant-interacting bacteria of the genera *Pseudomonas*, *Rhizobium* and *Sinorhizobium*. In addition, an inducible system was also developed to modulate pleD* expression and intracellular c-di-GMP rises on demand.

## Methods

### Bacteria and culture conditions

Bacteria and plasmids used in this work are listed in Additional file 1: Table S1*. E. coli* and *Pseudomonas* strains were grown routinely in Luria–Bertani broth (LB; containing 10 g/L tryptone, 5 g/L yeast extract, 5 g/L NaCl) at 37 °C or 28 °C respectively. Cultures of rizobial strains (Sme, Ret and Rle) were grown at 28 °C in TY broth (tryptone-yeast extract-CaCl_2_) [23] for Sme and Ret and YGT broth (glucose 15 g/L, tryptone 5 g/L, CaCl_2_ · 2H_2_O 0.6 g/L, yeast extract 2.5 g/L) for Rle. MM medium [24] was used for both rhizobial strains and Pto in different assays. When required, antibiotics were added at the following final concentrations: Tetracycline (Tc), 10 μg/ml for *E. coli*, Pto and Sme and 5 μg/ml for Ret and Rle; Kanamycin (Km) 50 μg/ml for all strains. All free-living cultures of strains carrying pJB3Tc19 or pJBpleD* plasmids contained Tc to prevent plasmid losses, except to evaluate the loss of plasmids without antibiotic pressure.

Stability of all mini-Tn*7* constructs was evaluated in all strains. Overnight cultures grown under Tc or Km selection were diluted 1/100 in nonselective LB (Pto), TY (Sme and Ret) or YGT (Rle) media, and incubated for 24 h at 28 °C with shaking. Several rounds of dilutions in nonselective media were repeated for at least 100 generations. After this, serial dilutions were spread on nonselective and selective agar plates, and CFUs (colony forming units) counted after incubation at 28 °C. Marker stability was determined as the ratio (%) of CFUs grown in selective medium out of the total CFUs appeared in nonselective plates.

### Construction and insertion of mini-Tn7 vectors into Gram-negative bacteria

The gene pleD* together with the *lac* promoter was PCR amplified from pJBpleD* vector [13] with pJB3Tc19-F and pleDTn7 primers. The fragment was cloned in pCR^®^^−^XL-TOPO^®^ and the resulting vector pTOPO-pleD* was digested with EcoRI and SacI. The insert was subcloned in pUC18T-mini-Tn*7*T (AY599230; [16] previously digested to give plasmid mini-Tn*7*pleD*. Kanamycin (Km^r^) or Tetracycline (Tc^r^) resistance cassettes from p34S-Km (AF062080) and p34S-Tc (AF062082), respectively, were introduced, after KpnI digestion, adjacent to the pleD* gene, obtaining mini-Tn*7*pleD*Km and mini-Tn*7*pleD*Tc, respectively. To obtain control strains without the gene pleD*, a NcoI internal deletion of 1114 bp of the 1380 bp of pleD* was performed, resulting in plasmids mini-Tn*7*Km and mini-Tn*7*Tc. Mini-Tn*7* plasmids containing the pleD* gene were maintained in *E. coli* β2155 (*lacI*^*q*^) [25] to prevent pleD* overexpression, whereas control plasmids with mini-Tn*7*Km and Tc plasmids were maintained in *E. coli* β2163 strain [25].

Triparental matings, as described in [26] were employed to deliver the mini-Tn*7* constructs into the genomes of *Pseudomonas syringae* pv. tomato DC3000 (Pto), *Sinorhizobium meliloti* 8530 (Sme), *Rhizobium etli* CFN42 (Ret) and *Rhizobium leguminosarum* bv. viciae UPM791 (Rle). *E. coli* β2163 bearing the pUX-BF13 plasmid carrying the transposase genes was used as helper strain for transposition.

### Motility assays

Motility assays were carried out as described in [13]. For swimming motility the strains were resuspended from MM plates and adjusted to an OD_600_ of 1. Two μl were spotted onto semisolid Bromfield medium (0.3 % agar) and halo diameter measured after incubation at 28 °C. Surface motility was analysed using a protocol previously described [27]. We used semisolid MM plates containing 0.6 % purified agar (Agar Noble, Difco), and a representative migration zone from one of the three biological replicates for each strain were imaged after 24–48 h at 28 °C for Pto, and 72 h at 28 °C for Ret and Rle.

### Congo red and calcofluor binding assays

To observe the production of exopolysaccharides, Sme, Ret and Pto strains were grown on solid MM plates supplemented with Congo red (CR; 125 μg/ml) or with calcofluor (CF; 200 μg/ml). Rle strains were grown on YGT media with the same concentration of CR and CF described above. Calcofluor binding was observed under UV light. CR and CF plates were photographed after 3 days incubation at 28 °C.

To quantify CF binding, 500 μl of a starting culture in rich broth was washed twice with MM and diluted 1/100 into 10 ml flasks containing MM supplemented with CF (100 μM). Flasks were incubated for 48 h at 28 °C (24 h at 20 °C for Pto). Afterwards, cultures were centrifuged and supernatants removed. The pellets were suspended in 2 ml distilled water and disposed in 24-well plates. Measures of three replicates from independent cultures for each strain were performed in a PTI fluorimeter (Photon Technology International).

### Biofilm assays

All strains were resuspended from a MM plate, washed with MM and diluted to a DO_600_ of 0.1. Aliquots of 200 μl were placed into the wells of sterile 96-well polystyrene plates (Sarstedt) and left in a humid chamber at 28 °C for 3 days. After incubation, the liquid from the wells was removed by aspiration and wells were washed with 240 μl of deionised water. 240 μl of Crystal Violet (CV; 0.1 % in water) was added to each well and left to stain for 1 h. The excess of crystal violet was removed by aspiration and each well was washed carefully with 240 μl of deionised water three times. 240 μl of 70 % ethanol was added to each well and the plate was gently agitated for at least 1 h. Ethanol suspension was diluted 1/2 for Ret and 1/7 for Rle for purple color quantification. Eight technical replicates from three separate cultures for each strain were measured at A550 nm in a Sunrise microplate reader (Tecan).

### Intracellular c-di-GMP measurements

c-di-GMP was extracted using a protocol described in [13]. Bacteria were grown in 10 ml of TY for Ret and Sme, YGT for Rle or LB broth for Pto. The area of the ion m/z 540 peak was used to estimate the amount of c-di-GMP in each sample. For quantification, a standard curve was established using synthetic c-di-GMP (Axxora) dissolved in ammonium acetate (10 mM pH 5.5) at a range of concentrations (20 nM, 200 nM, 2 μM and 20 μM). After subtracting the basal 250 nM spike, c-di-GMP concentrations in each strain culture were standardized with the total protein contents determined by Bradford assay [28]. Three biological replicates of each strain were measured and values were expressed as pmol c-di-GMP /mg protein ± standard error.

### Stability of constructs in symbiotic assays

Bean, vetch and alfalfa seeds (*Phaseolus vulgaris* cv. Contender, *Vicia sativa* cv. Jose and *Medicago sativa* cv. Aragon, respectively) were surface-sterilized and germinated as previously described [13, 29]. 12 bean or 25 vetch and alfalfa seedlings were sown in Leonard-type assemblies containing vermiculite:perlite (3:1) in the top part, and nitrogen-free nutrient solution [30] in the bottom. Each seedling was inoculated with 10^6^ CFU of the compatible bacterial symbiont (Ret for beans, Rle for vetch and Sme for alfalfa). Bean and alfalfa plants were cultivated in a growth chamber with 16/8-h light/dark photoperiod at 24/16 °C day/night and 75 % relative humidity. Vetch plants were grown in a greenhouse.

To test the stability in planta of the mini-Tn*7* constructs and plasmids pJB3Tc19 and pJBpleD*, fifty of the nodules formed by each strain were surface-sterilised with HgCl_2_ 0.25 % for 5 min followed by washing with abundant sterile deionised water. Nodules were individually crushed and the content spread on selective (Km or Tc) and nonselective plates. Plasmid stability was determined following the percentage of nodules containing bacteria able to grow in media with antibiotics.

### Quantitative RT-PCR assay

RNA extractions for real-time RT-PCR were carried out using the Qiagen RNeasy RNA purification kit (Qiagen) and following the manufacturer’s instructions. Total RNA (1 μg) treated with RNase-free Dnase I (Qiagen) was reverse-transcribed using Superscript II reverse transcriptase (Invitrogen) and random hexamers (Roche) as primers. Quantitative real-time PCR was performed with a iCycler iQ5 (Bio-Rad). Each 25 μl reaction contained 1 μl cDNA, 200 nM of each primer and iQ SyBrGreen Supermix (Bio-Rad). Control PCRs of the RNA samples were also performed to confirm the absence of contaminating genomic DNA. Samples were initially denatured by heating at 95 °C for 3 min, followed by a 35-cycle amplification and quantification programme (95 °C for 30 s, 60 °C for 30 s and 72 °C for 30 s). Melting curve analysis was conducted to ensure amplification of a single product. The efficiency of each primer pair (*E*) was determined by running 10-fold serial dilutions (four dilution series) of genomic DNA as template and generating a standard curve by plotting the log of the dilution factor against the *C*_T_ value during amplification of each dilution. Amplification efficiency was calculated using the formula [*E* = (10^(1/*a*)^ − 1) × 100], where *a* is the slope of the standard curve. The relative expression of pleD* gene was normalized to that of 16S rRNA gene, which was used as reference.

### Construction of a regulatory system of the pleD* expression in mini-Tn7 strains

The *lacI*^*q*^ gene was extracted by McsI digestion from the expression vector pQE-80L (Quiagen). The 1610 bp fragment was cloned into the pBBR1MCS5 [31] previously digested with SmaI. The resulting plasmid pBBRlacI^q^ was introduced into Sme Tn*7*pleD*Km strain using *E. coli* β2163 donor strain as described in [26].

## Results and discussion

### Construction and insertion of mini-Tn7 vectors to increase intracellular levels of c-di-GMP in Gram-negative bacteria

PleD from *Caulobacter crescentus* was one of the first and likely the best characterised DGC. It contains two N-terminal receiver domains which regulate DGC activity upon phosphorylation [32]. In contrast, PleD* is a constitutively active mutant variant of PleD with high DGC activity independent of its phosphorylation status, although it is still subject to feedback regulation through its allosteric I-site, avoiding deleterious effects for uncontrolled DGC activity in the cell [33, 34]. Plac promoter was selected to control pleD* expression since it behaves as a constitutive promoter in the absence of the LacI repressor. Plac has been used in multiple vectors, including different sets of Tn*7* constructs, designed to express a variety of genes in phylogenetically diverse gram-negative and gram-positive bacteria, e. g. *Pseudomonas aeruginosa*, *Xanthomonas campestris*, *Lactobacillus casei*, *Agrobacterium tumefaciens* or *Synechococcus elongatus* [35–42].

**Fig. 1:**
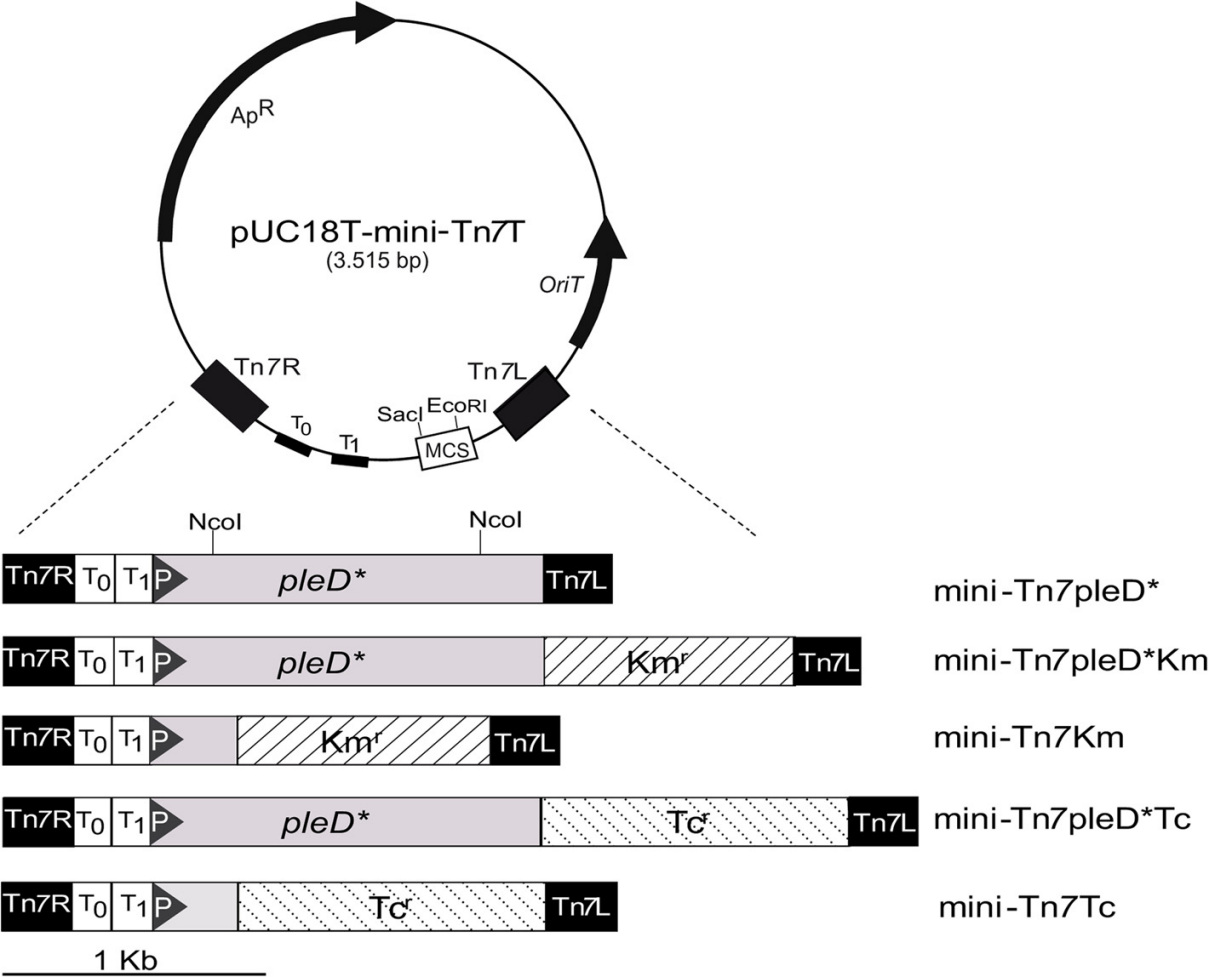
Mini-Tn*7* delivery constructions originated from pUC18T-mini-Tn*7*T suicide plasmid. The pleD* gene, under the *lac* promoter, was cloned between the Tn7 Left and Right ends. Downstream pleD* Tetracycline and Kanamycin resistance genes were cloned to facilitate selection. Vectors with resistance genes but without pleD*, were obtained after removing a NcoI internal fragment to pleD*. Ap^R^, Km^R^, Tc^R^ stand for resistance to Ampicillin, Kanamycin and Tetracycline, respectively; MCS, multi-cloning site; T_0_T_1_, transcriptional terminators from bacteriophage λ and *E. coli rrnB* operon, respectively; Tn*7*L and Tn*7*R, left and right ends of Tn*7* transposon, respectively; P, *lac* promoter

We initially constructed a mini-Tn*7* derivative containing the pleD* gene under the Plac control, mini-Tn*7*pleD* which carries no antibiotic selective markers (Fig. 1). This plasmid was maintained in strain *E. coli* β2155 (*lacI*^*q*^) [25] to prevent undesired PleD* expression in the donor. Triparental matings were performed to deliver the mini-Tn*7*pleD* plasmid into the genomes of *Pseudomonas syringae* pv. tomato DC3000 (Pto), *Sinorhizobium meliloti* 8530 (Sme), *Rhizobium etli* CFN42 (Ret) and *Rhizobium leguminosarum* bv. viciae UPM791 (Rle). *E. coli* β2163 bearing the pUX-BF13 plasmid [17] carrying the transposase genes was used as helper strain for transposition.

pleD* expression in a given strain can be easily followed, as it gives rise to colonies that usually stain red in plates supplemented with Congo Red (CR). We could isolate Ret transposants which formed strong red colonies in CR plates. Such red colonies appeared at a frequency of 10^−4^ (Table 1) and were easily distinguished amongst hundreds of non-coloured, wild-type colonies. However, the utility of this mini-Tn*7*pleD* construct without selective markers was limited to bacterial recipients, like *R. etli*, where transposition occurs at a high frequency and transposants display an easily selectable c-di-GMP-dependent phenotype. To facilitate selection of transposants, mini-Tn*7*pleD*Km and mini-Tn*7*pleD*Tc vectors, as well as derivatives with a pleD* deletion, were constructed (Fig. 1). Transposition efficiencies were relatively low albeit dependent on the bacterial recipient and also on the mini-Tn*7* version. In general, transposition efficiencies were higher with Km^r^ than with Tc^r^ constructs. Furthermore, transposition efficiencies of pleD*-deleted constructs were generally higher than their pleD* relatives (Table 1), suggesting that size of the mini-transposon can affect transposition efficiency, as proposed earlier [17].

**Table 1:** Transposition efficiencies of mini-Tn*7* constructs

Recipient strain	Mini-Tn*7*	Frequency^a^
*R. etli* CFN42 (Ret)	Tn*7*pleD*	3,0 x 10^−4^
	Tn*7*pleD*Km	6,5 x 10^−6^
	Tn*7*Km	5,2 x 10^−6^
	Tn*7*pleD*Tc	9,8 x 10^−8^
	Tn*7*Tc	5,1 x 10^−6^
*R. leguminosarum* bv. viciae UPM791 (Rle)	Tn*7*pleD*Km	2,5 x 10^−8^
	Tn*7*Km	4,8 x 10^−8^
	Tn*7*pleD*Tc	<10^−9^
	Tn*7*Tc	1,8 x 10^−9^
*S. meliloti* 8530 (Sme)	Tn*7*pleD*Km	4 x 10^−8^
	Tn*7*Km	8 x 10^−8^
	Tn*7*pleD*Tc	3,5 x 10^−9^
	Tn*7*Tc	2,3 x 10^−8^
*P. syringae* pv. tomato DC3000 (Pto)	Tn*7*pleD*Km	1,6 x 10^−7^
	Tn*7*Km	4,8 x 10^−5^
	Tn*7*pleD*Tc	7 x 10^−8^
	Tn*7*Tc	2 x 10^−5^

^a^Mini-Tn*7* frequency of transposition expressed as the number of transposants per input receptor cell

The location of the mini-Tn*7* insertions in each bacterial strain were determined by PCR and/or Southern hybridization. Pto carries a single copy of *glmS* and therefore a single *att*Tn*7* site, whereas Rle and Sme genomes have two genes with glucosamine-fructose-6-phosphate aminotransferase activity: *glmS* (chromosome) and *nodM* (Sym plasmid), both associated with *att*Tn*7* sites. On the other hand, two *glmS* genes, *glmS1* and *glmS2* have been annotated in the Ret CFN42 genome [43], however only *glmS1* seems to have an *att*Tn*7* site, according to known *att*Tn*7* sequences from different bacteria [19, 20, 22, 44, 45].

As expected, in Pto and Ret all transposition events were associated with the *att*Tn*7* site located downstream *glmS* and *glmS1*, respectively. In Rle all the transposants analyzed had the mini-Tn*7* insertions downstream the *glmS* gene and none were linked to *nodM*. In contrast, in Sme *nodM* was by far the preferred site of insertion, and only 2 % of the mini-Tn*7* insertions were associated to *glmS*, in agreement with previous reports [46]. This is noteworthy, since *nodM* is part of the *nodMnolFGnodN* operon and *nodM*-associated insertions result in undesired polar effects, leading to reduced nodulation efficiency [47, 48]. This site preference, added to the low efficiency of transposition, determined that we could only isolate *glmS* Km^r^, but not Tc^r^, transposants in Sme.

### c-di-GMP intracellular levels in mini-Tn7pleD* transposants

**Fig. 2:**
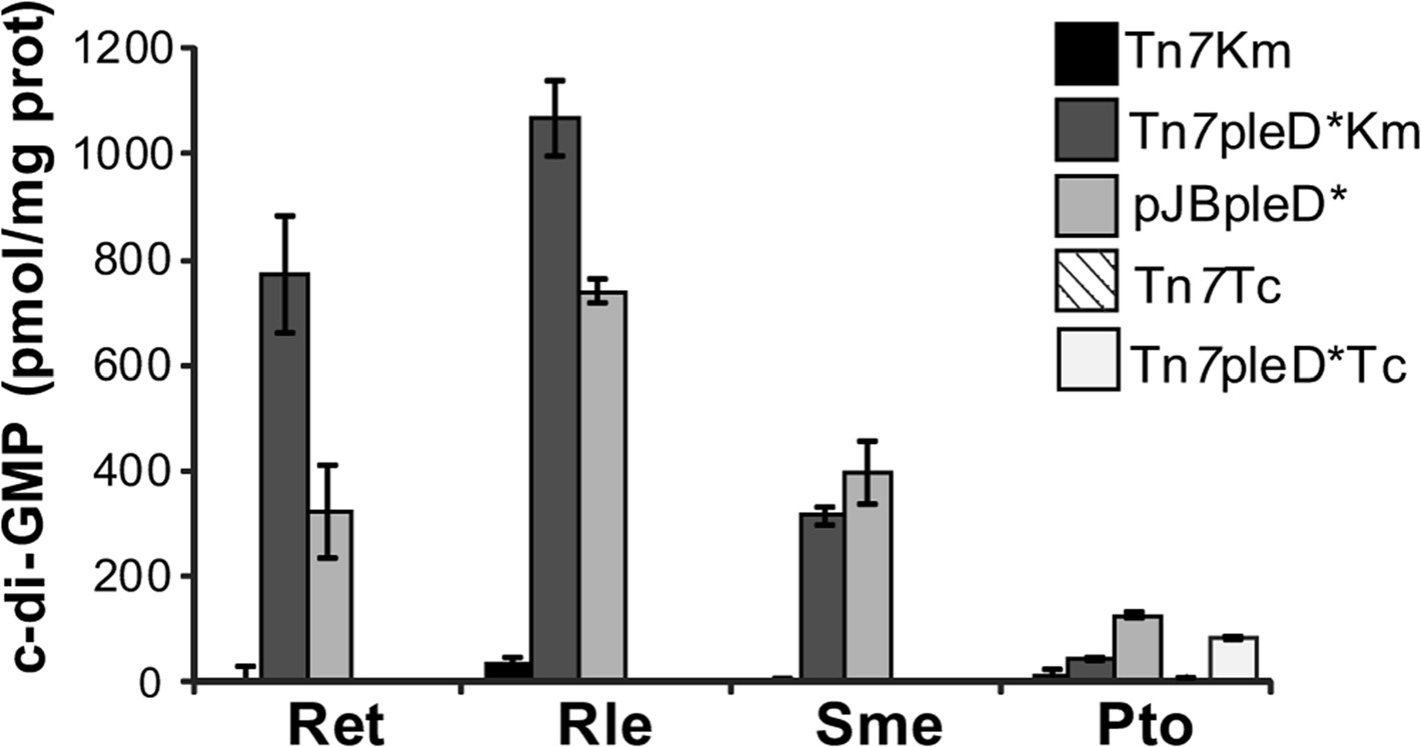
Quantification of intracellular c-di-GMP levels. c-di-GMP contents of *Rhizobium etli* CFN42 (Ret), *Sinorhizobium meliloti* 8530 (Sme), *Rhizobium leguminosarum* bv. viciae UPM791 (Rle) and *Pseudomonas syringae* pv. tomato DC3000 (Pto), with plasmid-encoded (pJBpleD*) or chromosomally integrated pleD* gene. Tc transposants in rhizobial strains displayed comparable phenotypes to Km and their c-di-GMP levels were not determined. Control strains without pleD* carried mini-Tn7Km or mini-Tn7Tc. Standard error of three biological replicates are shown

Intracellular c-di-GMP levels of representative mini-Tn*7*pleD* transposants were quantified and compared with strains carrying the pJBpleD* plasmid and control strains (Fig. 2). Since Km and Tc transposants displayed comparable phenotypes (see below), we chose to measure the Tn*7*pleD*Km transposants as representatives for the rhizobial strains. The levels of wild type rhizobial strains were near the detection limit of the technique used [13]. However, the mini-Tn*7*pleD*Km transposants showed significantly higher c-di-GMP levels than their respective controls (Tn*7*Km) in all species, with similar or even higher values than the corresponding derivatives carrying pJBpleD* plasmid (Fig. 2). However, a modest c-di-GMP increment was observed in the Pto Tn*7*pleD*Km transposant, which showed a three-fold increase above the wild-type levels but three-fold lower levels than the Pto pJBpleD*. In contrast, a Pto Tn*7*pleD*Tc transposant showed two-fold higher c-di-GMP levels than the Tn*7*pleD*Km transposant (Fig. 2). The differences between these transposants were probably due to different pleD* expression levels, since the Pto Tn*7*pleD*Km transposant had 3.7 fold lower pleD* transcripts levels than the Pto Tn*7*pleD*Tc strain (as measured by qRT-PCR). Although we cannot offer an explanation for these differences, the results suggest the convenience of analyzing several independent transposants with regard to phenotypic changes and c-di-GMP intracellular levels.

### Effects of mini-Tn7pleD* insertions on bacterial free-living phenotypes

Raising c-di-GMP levels usually leads to a number of phenotypic changes related to colony morphology, e.g., overproduction of cellulose and other EPSs, and motility reduction in different bacteria [13].

**Fig. 3:**
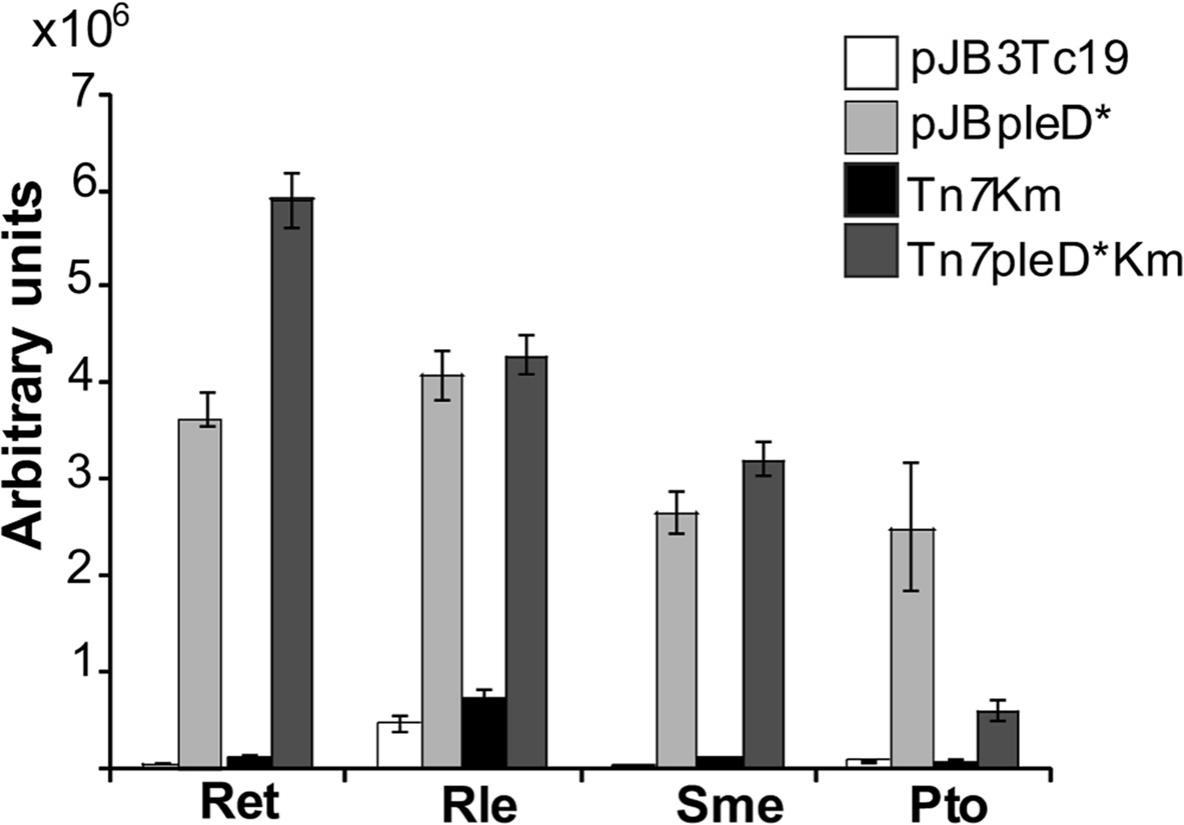
Quantification of calcofluor-derived fluorescence of *R. etli* CFN42 (Ret), *R. leguminosarum* bv. viciae *UPM791* (Rle), *S. meliloti* 8530 (Sme), and *P. syringae* pv.tomato DC3000 (Pto) expressing plasmid-encoded (pJBpleD*) or chromosomally integrated pleD* gene, and their respective control strains. Mean values from three independent cultures ± standard deviation

Similar to plasmid pJBpleD*, all mini-Tn*7*pleD* generated Congo Red (CR^+^) and Calcofluor (CF^+^) phenotypes (Additional file 1: Figure S1), with an enhanced CF-derived fluorescence (Fig. 3). These phenotypes were not observed in non-pleD* strains (Fig. 3 and Additional file 1: Figure S1). CR binds to D-glucopyranosyl units, basic or neutral polysaccharides, as well as to some proteins, whereas CF binds to β(1–4) and β(1–3) glycosidic bonds of polysaccharides [49]. In Pto, Ret and Rle the CR^+^ and CF^+^ stainings are likely due to overproduction of cellulose [13], whereas in *S. meliloti* 8530 this is due to another CR^+^/CF^+^ related polymer, a recently described mixed-linkage β-glucan [15]. Stronger CR and CF stainings were observed for the Pto Tn*7*pleD*Tc than for the Pto Tn*7*pleD*Km strain, in agreement with the higher c-di-GMP intracellular and pleD* expression levels in that transposant, as described above (Fig. 2 and Additional file 1: Figure S1).

Correlating with enhanced polysaccharide production, all strains expressing pleD* showed a strong aggregative behaviour, forming flocs in liquid media (data not shown). Enhanced polysaccharide production usually leads to biofilm formation [50–52]. The pleD* expression, either from plasmid or from integrated mini-Tn*7*pleD* constructs, similarly led to an enhanced biofilm formation, which was quantified in Ret and Rle by Cristal Violet staining in microtiter plates (Additional file 1: Figure S2). Pto and Sme, on the other hand, formed air-liquid interface biofilms, pellicles that easily collapsed and could not be retained during the CV staining procedures, hindering quantification [13].

High c-di-GMP levels usually inhibit bacterial motility [53–57]. As expected, mini-Tn*7*pleD* transposants displayed strongly reduced swarming and swimming motilities in all strains (Additional file 1: Figure S3), in a similar way to what has been reported for pJBpleD*-carrying bacteria [13].

### Stability of mini-Tn7pleD* insertions

Stability of mini-Tn*7* insertions was determined after 100 generations free-living growth in rich broth without antibiotic selective pressure and compared with the plasmid pJBpleD* or the empty vector pJB3Tc19. As reported previously [13] the pJB3Tc19 and especially the pJBpleD* plasmids were rapidly lost in the absence of Tc, particularly in Ret and Sme with less than 0.6 and 3.1 %, respectively, of Tc^r^ CFUs retaining pJBpleD* after 100 generations (Table 2). In stark contrast, all mini-Tn*7* insertions were 100 % stable in all strains tested. The instability of pleD* plasmid under non selective pressure could also be observed for free-living phenotypes (Additional file 1: Figure S4). For instance, strain Ret pJBpleD* under non selective conditions showed a progressive loss of the CR binding capacity, which gave rise to a segmented-colony phenotype after 5 days of growth, with most cells at the colony edges showing a CR^−^ phenotype (Additional file 1: Figure S4b). This was not observed with this strain in the presence of Tc. In contrast, the transposant Ret Tn*7*pleD*Tc strain did not show the colony sectored appearance, either in the presence or in the absence of Tc (Additional file 1: Figure S4b). Likewise, Ret Tn*7*pleD*Tc strain displayed a complete arrest of swimming motility either in the presence or absence of tetracycline, whereas strain Ret pJBpleD* showed reduced (but not fully arrested) swimming, and formed swimming haloes which were significantly larger in media without antibiotic pressure, evidencing loss of the pJBpleD* plasmid (Additional file 1: Figure S4a).

**Table 2:** Stability of plasmids and mini-Tn*7* insertions in different strains

Strain	Free-living stability^a^	Stability in symbiosis^b^
Ret pJB3Tc19	8,6 %	98 %
Ret pJBpleD*	0.6 %	0 %
Ret Tn*7*Km	100 %	100 %
Ret Tn*7*pleD*Km	100 %	90 %
Ret Tn*7*Tc	100 %	n.d.
Ret Tn*7*pleD*Tc	100 %	n.d.
Rle pJB3Tc19	73,5 %	86 %
Rle pJBpleD*	67,1 %	70 %
Rle Tn*7*Km	100 %	100 %
RleTn*7*pleD*Km	100 %	100 %
Rle Tn*7*Tc	100 %	n.d.
Rle Tn*7*pleD*Tc	n.d	n.d.
Sme pJB3Tc19	2,8 %	51 %
Sme pJBpleD*	3,1 %	22 %
Sme Tn*7*Km	100 %	100 %
Sme Tn*7*pleD*Km	100 %	91 %
Sme Tn*7*Tc	100 %	n.d.
Sme Tn*7*pleD*Tc	100 %	n.d.
Pto pJB3Tc19	100 %	n.d.
Pto pJBpleD*	76,8 %	n.d.
Pto Tn*7*Km	100 %	n.d.
Pto Tn*7*pleD*Km	100 %	n.d.
Pto Tn*7*Tc	100 %	n.d.
Pto Tn*7*pleD*Tc	100 %	n.d.

^a^Bacteria were grown in rich medium without antibiotics for at least 100 generations; the stability was determined as the ratio of CFU recovered on selective medium out of the total CFU obtained in nonselective medium

^b^Percentage of nodules containing bacteria that kept antibiotic resistance (for more details see supporting information)

n.d., not determined

The stability of mini-Tn*7* constructs in different rhizobial strains were also evaluated *in planta* (Table 2). Bacteria which kept the Tn*7*pleD*Km could be recovered from 90 to 100 % of root nodules. In contrast, the maintenance of pJBPleD* plasmid in nodules was significantly lower, being negligible or even undetectable in some cases (Table 2). This emphasizes the utility of our mini*-*Tn*7*pleD* constructs under experimental conditions where antibiotic selection is not feasible. Nevertheless, stability of mini-Tn*7*pleD*Km insertions in Sme and Ret seemed slightly lower in planta (90 %) than in free-living conditions (100 %; Table 2), indicating that during nodule infection there is a strong pressure against bacteria expressing high c-di-GMP levels, as suggested earlier [13].

### Modulation of pleD* expression in mini-Tn7 strains

Overexpression of DGCs usually have a deep impact in the c-di-GMP economy, generating strong phenotypes [58–60]. In that sense, modulating DGC expression in these mini-Tn*7*pleD* transposants could be useful in order to raise the intracellular levels of c-di-GMP “on-demand”. Since pleD* transcription is under the control of *lac* promoter, we evaluated if pleD* expression could be modulated in these mini-Tn*7* transposants using a *lacI*^*q*^-based system and the inducer Isopropyl β-D-1-thiogalactopyranoside (IPTG). In order to keep the pleD* expression at a minimum under non inducible conditions, we cloned the repressor *lacI*^*q*^ gene version from pQE-80L into the broad host range plasmid pBBR1MCS-5 [31], obtaining pBBRlacI^q^. This plasmid was introduced by conjugation in the Sme Tn*7*pleD*Km strain. pleD* expression, c-di-GMP levels and EPS production were determined in the absence and in the presence of IPTG (Table 3). qRT-PCR confirmed that pleD* was strongly repressed in the presence of LacI (Tn*7*pleD*Km pBBRlacI^q^) and absence of inducer. This repression state was alleviated by 1 mM IPTG. However, even with this high amounts of inducer, pleD* transcription did not reach the levels attained in the absence of the LacI repressor. The intracellular levels of c-di-GMP followed the same trend (Table 3). 1 mM of IPTG generated a 3-fold increase of c-di-GMP levels in strain Sme Tn*7*pleD*Km pBBRlacI^q^, just half of the rise achieved in the absence of the repressor (Sme Tn*7*pleD*Km pBBR1MCS5). However, this addition of IPTG was enough to produce similar impacts on EPS production, increasing it up to 1 Log (Table 3 and Additional file 1: Figure S5), indicating that maximum EPS production can be achieved at intermediate c-di-GMP levels. Overall these results demonstrate that the *lacI*^q^/IPTG system could be useful to modulate the c-di-GMP intracellular levels generated by the mini-Tn7pleD* insertions. Even when a complete de-repression of pleD* by IPTG could not be achieved, c-di-GMP levels were sufficiently high to generate the expected phenotypes.

**Table 3:** Efficacy of the *lacI* ^q^-IPTG system to modulate pleD* expression

Strain	Relative pleD* expression^1^		Intracellular c-di-GMP ^2^		EPS production^3^	
	IPTG -	IPTG +	IPTG -	IPTG +	IPTG -	IPTG +
Sme Tn*7*pleD*Km pBBR1MCS5	15,11 ± 1,98	n.d.	317,16 ± 13,29	n.d.	2,66 × 10^6^ ± 6,9 × 10^4^	2,64 × 10^6^ ± 7,34 × 10^4^
Sme Tn*7*pleD*Km pBBRlacI^q^	1,00 ± 0,26	6,08 ± 1,06	55,10 ± 16,27	152,05 ± 8,50	3,51 × 10^5^ ± 1,19 × 10^4^	2,23 × 10^6^ ± 6,36 × 10^4^
Sme Tn*7*Km	-	-	-	n.d.	6,20 × 10^4^ ± 5,54 × 10^3^	7,39 × 10^4^ ± 3,44 × 10^3^

^1^Relative expression (fold change) to Sme Tn*7*pleD*Km pBBRlacI^q^ strain without IPTG (repression state) by qRT-PCR. In all strains pleD* expression was normalized with to 16S rRNA levels

^2^pmol of c-di-GMP/mg of total protein. The c-di-GMP levels of Sme Tn7Km is under the technical limit of detection

^3^CF-derived Fluorescence (arbitrary units)

n.d., not determined

## Conclusions

In this work, we have constructed a set of mini-Tn7 vehicles to allow integration and stable expression of a DGC gene, pleD*, useful to artificially increase the intracellular levels of the second messenger c-di-GMP in bacteria. The utility of these new mini-Tn*7*pleD* tools has been proved in several α- and γ-proteobacteria, where significant increases in intracellular c-di-GMP contents are achieved. As a consequence, phenotypic changes such as enhanced polysaccharide production and biofilm formation, and reduced motility are easily observed. Nonetheless, a careful interpretation of the bacterial phenotypes generated by the high non-physiological pleD*-dependent c-di-GMP levels should be exercised. The highly stable mini-Tn*7*pleD* constructs are particularly convenient under conditions where a selective pressure cannot be applied to ensure DGC expression, e.g., during interaction with an eukaryotic host. Furthermore, we have also implemented an inducible system to modulate pleD* expression and intracellular c-di-GMP rises on demand.

## Additional file

Additional file 1:
**Table S1.** Bacterial strains and plasmids used in this work. **Table S2.** Primers used in this work. **Figure S1.** Congo red (CR) and calcofluor (CF) staining of* R. etli* CFN42 (Ret), *S. meliloti* 8530 (Sme), *R. leguminosarum* bv. viciae UPM791 (Rle) and *P. syringae* pv.tomato DC3000 (Pto) expressing *pleD** in mono and multicopy and their respectives control strains.** Figure S2.** Biofilm formation by *R. etli* CFN42 (Ret) and *R. leguminosarum* bv. viciae UPM791 (Rle) strains expressing *pleD** in multicopy (pJBpleD*), monocopy (Tn7pleD*Km) and the control strain without *pleD** (Tn7Km). **Figure S3.** Motility reduction in mini-Tn7pleD* strains. **Figure S4.** Stability of mini-Tn7pleD* in *R. etli* CFN42 (Ret) under non selective conditions. **Figure S5.** Control expression of pleD* by *lacI* ^q^/IPTG system. Colony morphology of *S. meliloti* (Sme) after grown for two days on MM plates supplemented with Congo Red (CR) with and without the inducer IPTG (1mM). (PDF 2631 kb)
