# Supplementary material for: Mini-Tn7 vectors for stable expression of diguanylate cyclase PleD* in Gram-negative bacteria
Source: BMC Microbiol. 2015 Sep 29;15:190. doi: 10.1186/s12866-015-0521-6 (PMC4587759; doi:10.1186/s12866-015-0521-6)
Supplement: Additional file 1: — Table S1. Bacterial strains and plasmids used in this work. Table S2. Primers used in this work. Figure S1. Congo red (CR) and calcofluor (CF) staining of R. etli CFN42 (Ret), S. meliloti 8530 (Sme), R. leguminosarum bv. viciae UPM791 (Rle) and P. syringae pv.tomato DC3000 (Pto) expressing pleD* in mono and multicopy and their respectives control strains. Figure S2. Biofilm formation by R. etli CFN42 (Ret) and R. leguminosarum bv. viciae UPM791 (Rle) strains expressing pleD* in multicopy (pJBpleD*), monocopy (Tn7pleD*Km) and the control strain without pleD* (Tn7Km). Figure S3. Motility reduction in mini-Tn7pleD* strains. Figure S4. Stability of mini-Tn7pleD* in R. etli CFN42 (Ret) under non selective conditions. Figure S5. Control expression of pleD* by lacI q/IPTG system. Colony morphology of S. meliloti (Sme) after grown for two days on MM plates supplemented with Congo Red (CR) with and without the inducer IPTG (1mM). (PDF 2631 kb) [file 12866_2015_521_MOESM1_ESM.pdf]

Additional files

Additional Tables:

Table S1. Bacterial strains and plasmids used in this work

Strains	Relevant characteristics	Reference
**Rhizobial strains**		
R. etli CFN42	Wild type	[1]
Ret Tn*7*Km	CFN42 mini-Tn*7*Km, Km^R	This work
Ret Tn*7*pleD*Km	CFN42 mini-Tn*7*pleD*Km, Km^R	This work
Ret Tn*7*Tc	CFN42 mini-Tn*7*Tc, Tc^R	This work
Ret Tn*7*pleD*Tc	CFN42 mini-Tn*7*pleD*Tc, Tc^R	This work
Ret Tn*7*pleD*	CFN42 mini-Tn*7*pleD*	This work
R. leguminosarum bv. viciae UPM791	Wild type, Sm^R	[2]
Rle Tn*7*Km	UPM791 mini-Tn*7*Km, Km^R	This work
Rle Tn*7*pleD*Km	UPM791 mini-Tn*7*pleD*Km, Km^R	This work
Rle Tn*7*Tc	UPM791 mini-Tn*7*Tc, Tc^R	This work
S. meliloti 8530	ExpR$^+$ derivative of Rm1021, Sm^R	[3]
Sme Tn*7*Km	8530 mini-Tn*7*Km, Km^R	This work
Sme Tn*7*pleD*Km	8530 mini-Tn*7*pleD*Km, Km^R	This work
Sme Tn*7*Tc	8530 mini-Tn*7*Tc, Tc^R	This work
Sme Tn*7*pleD*Tc	8530 mini-Tn*7*pleD*Tc, Tc^R	This work
**Pseudomonas strains**		
P. syringae pv. tomato DC3000	Rif^R	[4]
Pto Tn*7*Km	DC3000 mini-Tn*7*Km, Km^R	This work
Pto Tn*7*pleD*Km	DC3000 mini-Tn*7*pleD*Km, Km^R	This work
Pto Tn*7*Tc	DC3000 mini-Tn*7*Tc, Tc^R	This work
Pto Tn*7*pleD*Tc	DC3000 mini-Tn*7*pleD*Tc, Tc^R	This work
***E. coli* strains**		
TOP10	*F$^-$ mcr*A Δ*(mrr-hsd*RMS-*mcr*BC*) Φ80lacZ*ΔM15 Δ*lac*X74 *rec*A1, *ara*D139, Δ*(ara-leu)7697, galU, galK, rpsL(Str^r) end*A1,*nupG λ-*	Invitrogen®
β2163	*MG1655::ΔdapA::(erm-pir)RP4-2,Tc::Mu, Km^r, Em^r*	[5]
β2155	*RP4-2-Tc::Mu ΔdapA::(erm-pir) thrB1004, pro, thi, strA, hsdS, lacZ ΔM15, (F′ lacZ ΔM15,lacIq, traD36, proA+, proB+) Km^r, Sm^r, Em^r*	[5]
**Plasmids**		
pJB3Tc19	IncP , cloning vector Ap^R, Tc^R	[6]
pJBpleD*	pJB3Tc19 bearing *pleD** gene	[7]
pCR®-XL-TOPO®	Cloning vector, Km^R	Invitrogen®
pTOPO-pleD*	pCR®-XL-TOPO® with a 1784 bp fragment containing *pleD**, Km^R	This work
pUC18T-mini-Tn*7*T	pUC18 with mini-Tn*7* transposon, Ap^R,	[8]

mini-Tn7pleD*	pUC18T-mini-Tn7T with the EcoRI/SacI fragment containing *pleD** Ap^R,	This work
mini-Tn7pleD*Km	mini-Tn7pleD* with 1.2 Kb KpnI fragment containing Km marker Ap^R, Km^R,	This work
mini-Tn7Km	mini-Tn7pleD*Km with a 1114 bp NcoI internal deletion of *pleD** Ap^R, Km^R,	This work
mini-Tn7pleD*Tc	mini-Tn7pleD* with 1.3 Kb KpnI fragment containing Tc marker Ap^R, Tc^R	This work
mini-Tn7Tc	mini-Tn7pleD*Tc with a 1114 bp NcoI internal deletion, Ap^R, Tc^R	This work
pUX-BF13	Helper plasmid providing the Tn7 transposition functions in trans, Ap^R, mob+, ori-R6K	[9]
p34S-Tc	Plasmid containing a Tc marker, Tc^R	[10]
P34S-Km	Plasmid containing a Km marker, Km^R	[10]
pQE-80L	Expression vector, Ap^R	Invitrogen®
pBBR1MCS-5	Cloning vector, Gm^R	[11]
pBBRlacI^q	pBBR1MCS-5 with a McsI 1610 bp fragment containing the *lacI^q* gene, Gm^R	This work

Table S2. Primers used in this work.

Name	Sequence 5´-3´	Used in
pJB3Tc19-F	GCCTCTTCGCTATTACGCC	*pleD** amplification from pJBpleD*
pleDTn7	GAGCTCACGCAAACCGCCTCTCC	
pTn7L	ATTAGCTTACGACGCTACACCC	Verification of insertion under *glmS* region
pTn7R	CACAGCATAACTGGACTGATTTC	
glmS1etF	CCTGTTATCGTCATTGCTCC	Verification of insertion in Ret under *glmS*1 region
glmS1etR	CGACAGCAATCAGCAGGC	
glmS_pstF	TGGCGAACTCAAACACGG	Verification of insertion in Pto under *glmS* region
glmS_pstR	TACCGAGTAGAACCTCCTTAGC	
glmS_legF	CCTGTCATCGTCATCGCC	Verification of insertion in Rle under *glmS* region
glmS_legR	GCACGACGGCGATCAGC	
glmS_rmF	CCACGCCGAAGGTTACG	Verification of insertion in Sm under *glmS* region
glmS_rmR	AGGCTCGTTGCGGAACC	
Rm_NodM	GCGAGGTCAGTGTAGAACG	Verification of insertion in Sm under *nodM* region
RT_pleDF	AATGTCCGCCTGCTTGA	*pleD** expression by qRT-PCR
RT_pleDR	CAGAATGATGTCGGGCAG	
Rm 16S F	TCTACGGAATAACGCAGG	16S rRNA gene of Sme for qRT-PCR
Rm 16S R	GTGTCTCAGTCCCAATGT	
F-RT-16S	ACACCGCCCGTCACACCA	16S rRNA gene of Pto for qRT-PCR
R-RT-16S	GTTCCCCTACGGCTACCTT	

Additional Figures

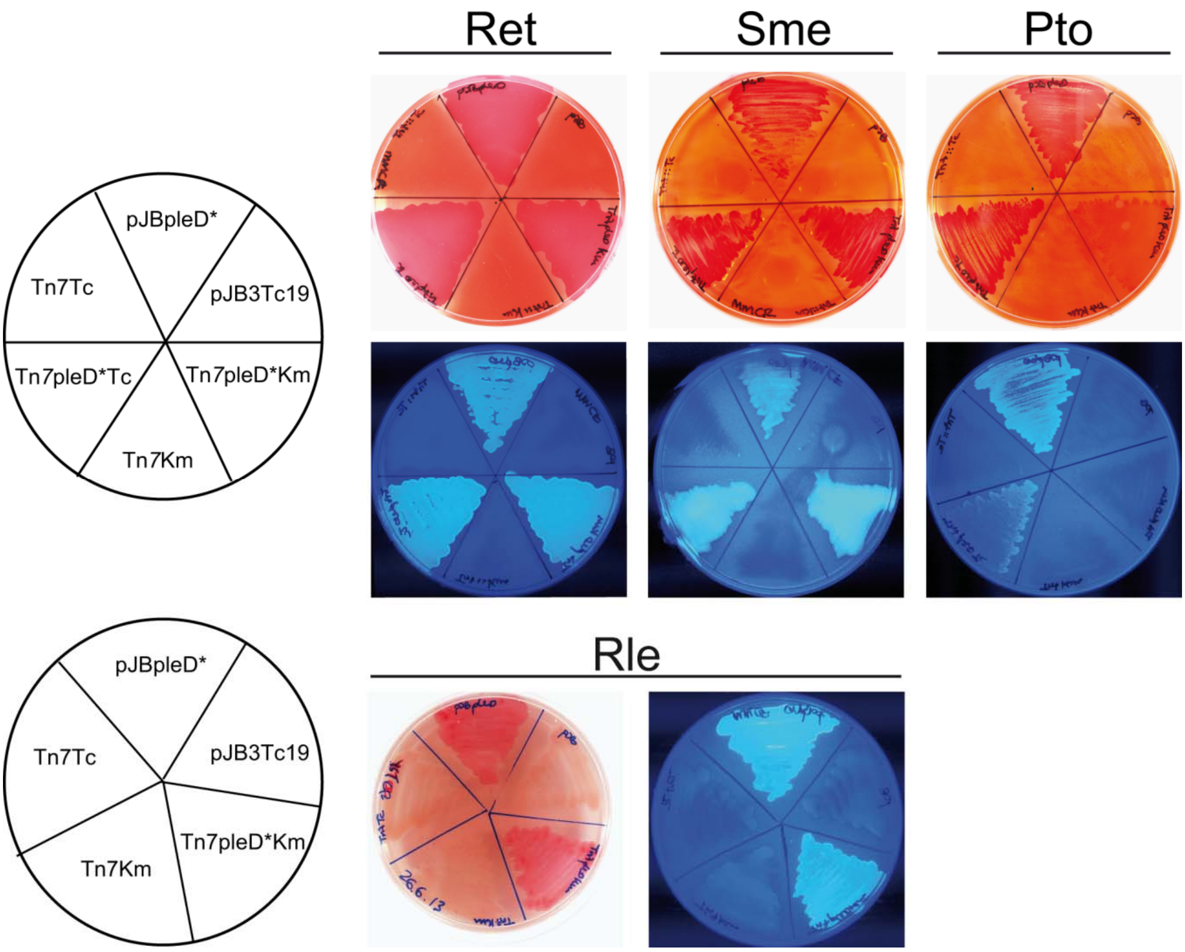

Figure S1. Congo red (CR) and calcofluor (CF) staining of *R. etli* CFN42 (Ret), *S. meliloti* 8530 (Sme), *R. leguminosarum* bv. viciae UPM791 (Rle) and *P. syringae* pv.tomato DC3000 (Pto) expressing *pleD** in single and multiple copies and their respectives control strains. Bacteria were grown on solid YGT, for Rle, or MM plates, for the rest of the strains, supplemented with congo red (CR; 125 µg/ml) or with calcofluor (CF; 200 µg/ml). Calcofluor binding was observed under UV light. CR and CF plates were photographed after 3 days incubation at 28ºC for rhizobial strains and for *Pseudomonas*.

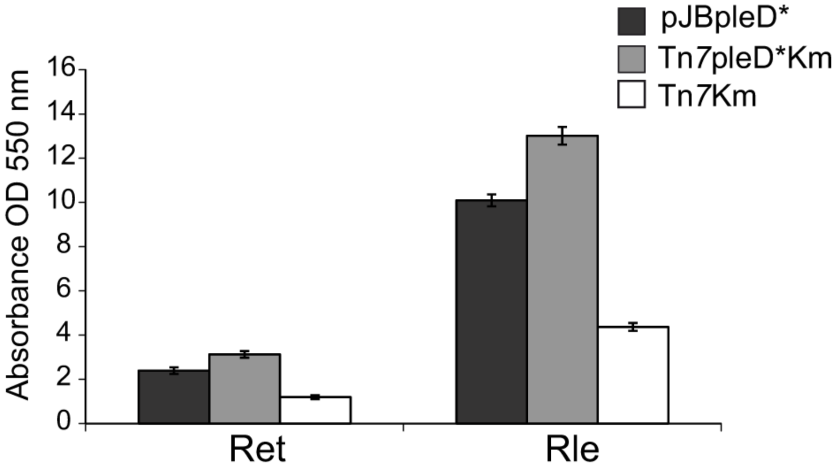

Figure S2. Biofilm formation by *Rhizobium etli* CFN42 (Ret) and *Rhizobium leguminosarum* bv. viciae UPM791 (Rle) strains expressing *pleD** in multicopy (pJBpleD*), single copy (Tn*7*pleD*Km) and the control strain without *pleD** (Tn*7*Km). Quantification of biofilm formation by crystal violet (CV) after 72h of growth in MM in a 96-well plate at 28 ºC. Bars represent the mean of eight wells from three biologial replicates ± standard error.

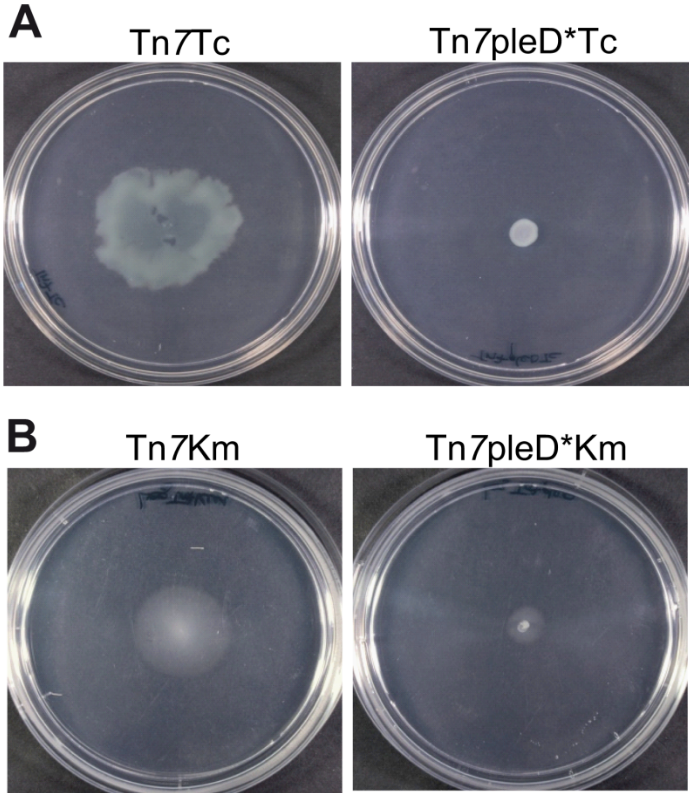

F**igure S3. Motility reduction in mini-Tn*7*pleD* strains.** (A) Surface motility of Pto strains after 24h at 28ºC onto semisolid MM plates (see methods). B) Swimming motility of Rle after 72h growing at 28 ºC. Pictures show a representative migration zone of each strain. At least three motility plates from three independent cultures per strain were used.

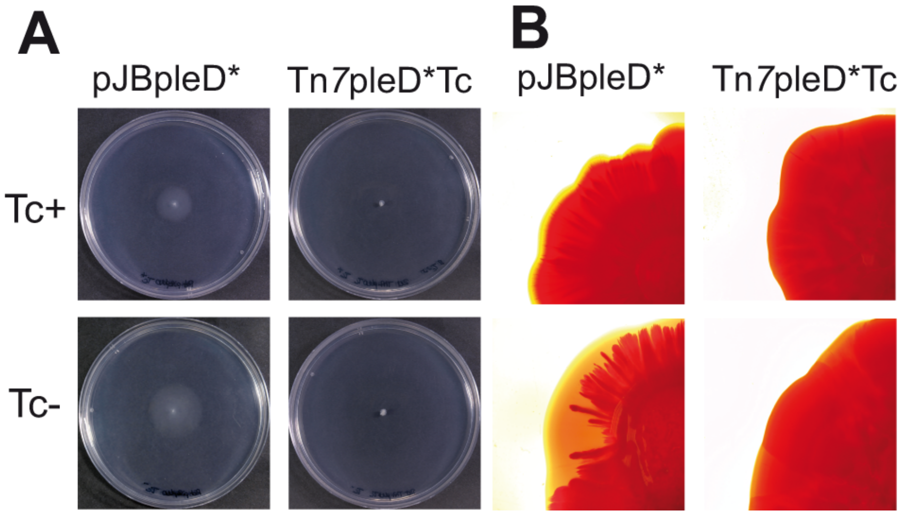

Figure S4. Stability of mini-Tn*7*pleD* in *Rhizobium etli* CFN42 (Ret) under non selective conditions. Ret strains with a plasmid-encoded (pJBpleD*) or chromosomally integrated *pleD** gene (Tn*7*pleD*Tc), were assayed for A) swimming motility or B) exopolysaccharide production by Congo Red (CR) staining in the presence and absence of tetracycline. (A) Swimming plates were imaged after 120 h at 28 ºC in semisolid Bromfield medium. (B) Colonies imaged after growth on solid MM plates supplemented with CR; 125 µg/ml, at 28ºC for 120 h. Representative pictures of at least three different plates from three independent cultures of each strain are shown.

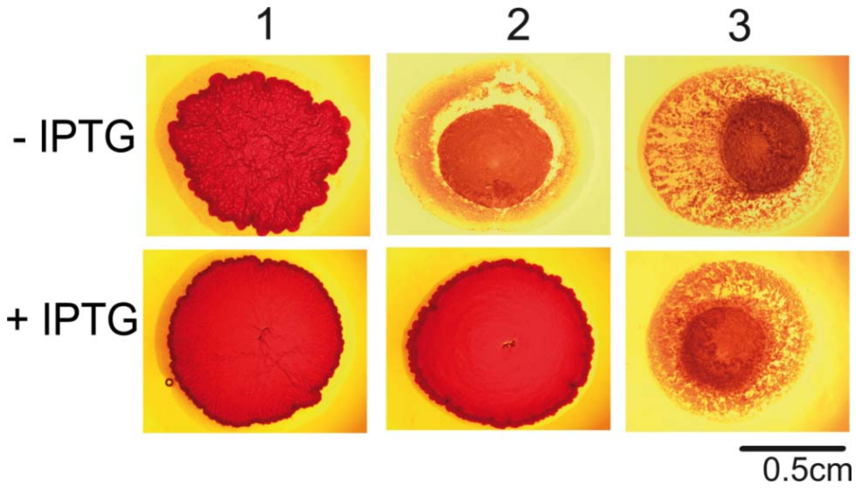

Figure S5. Control expression of *pleD by *lacI*^{q}/IPTG.** Colony morphology of *S. meliloti* (Sme) after two days growth on MM plates supplemented with Congo Red (CR), with and without the inducer IPTG (1mM): Sme Tn*7*pleD*Km pBBR1MCS5 (1), Sme Tn*7*pleD*Km pBBRlacI^{q} (2) and Sme Tn*7*Km (3). Scale bar is depicted. Representative pictures of at least three different plates from three independent cultures of each strain are shown.

References of additional material

1. Quinto C, De L, V, Flores M, Leemans J, Cevallos MA, Pardo MA, Azpiroz R, De Lourdes GM, Calva E, Palacios R: **Nitrogenase reductase: A functional multigene family in *Rhizobium phaseoli***. *ProcNatlAcadSciUSA* 1985, **82**(4):1170-1174.
2. Leyva A, Palacios JM, Mozo T, Ruiz-Argueso T: **Cloning and characterization of hydrogen uptake genes from *Rhizobium leguminosarum***. *JBacteriol* 1987, **169**(11):4929-4934.
3. Pellock BJ, Teplitski M, Boinay RP, Bauer WD, Walker GC: **A LuxR homolog controls production of symbiotically active extracellular polysaccharide II by *Sinorhizobium meliloti***. *Journal of bacteriology* 2002, **184**(18):5067-5076.
4. Cuppels DA: **Generation and Characterization of Tn5 Insertion Mutations in *Pseudomonas syringae* pv. tomato**. *ApplEnvironMicrobiol* 1986, **51**(2):323-327.
5. Demarre G, Guerout AM, Matsumoto-Mashimo C, Rowe-Magnus DA, Marliere P, Mazel D: **A new family of mobilizable suicide plasmids based on broad host range R388 plasmid (IncW) and RP4 plasmid (IncPalpha) conjugative machineries and their cognate *Escherichia coli* host strains**. *ResMicrobiol* 2005, **156**(2):245-255.
6. Blatny JM, Brautaset T, Winther-Larsen HC, Haugan K, Valla S: **Construction and use of a versatile set of broad-host-range cloning and expression vectors based on the RK2 replicon**. *ApplEnvironMicrobiol* 1997, **63**(2):370-379.
7. Pérez-Mendoza D, Aragon IM, Prada-Ramírez HA, Romero-Jiménez L, Ramos C, Gallegos MT, Sanjuán J: **Responses to elevated c-di-GMP levels in mutualistic and pathogenic plant-interacting bacteria**. *PloS one* 2014, **9**(3):e91645.
8. Choi KH, Gaynor JB, White KG, Lopez C, Bosio CM, Karkhoff-Schweizer RR, Schweizer HP: **A Tn*7*-based broad-range bacterial cloning and expression system**. *NatMethods* 2005, **2**(6):443-448.
9. Bao Y, Lies DP, Fu H, Roberts GP: **An improved Tn*7*-based system for the single-copy insertion of cloned genes into chromosomes of gram-negative bacteria**. *Gene* 1991, **109**(1):167-168.
10. Dennis JJ, Zylstra GJ: **Plasposons: modular self-cloning minitransposon derivatives for rapid genetic analysis of gram-negative bacterial genomes**. *ApplEnvironMicrobiol* 1998, **64**(7):2710-2715.
11. Kovach ME, Elzer PH, Hill DS, Robertson GT, Farris MA, Roop RM, Peterson KM: **Four new derivatives of the broad-host-range cloning vector pBBR1MCS, carrying different antibiotic-resistance cassettes**. *Gene* 1995, **166**(1):175-176.
